# Chicago Dental Society

**Published:** 1866-04

**Authors:** 


### CHICAGO DENTAL SOCIETY.

At the Annual Meeting of the Chicago Dental Society held April 2d 1866 the following officers were elected, viz;

Dr. J. W. Ellis, President.
" M. W. Sherwood, 1st Vice President.
" S. B. Noble, 2d Vice President.
" J. C. Dean, Cor. and Recording Secretary.
" Wm. Albaugh, Treasurer.
" W. A. Stevens, Librarian.
" M. S. Dean, }
" W. C Dyer, } Executive Committee.
" A. J. Harris, }

